# Baseline characteristics of a diagnostically defined early psychosis cohort entering supported employment and education: findings from the SEEearly trial

**DOI:** 10.1007/s00406-026-02236-8

**Published:** 2026-04-13

**Authors:** A. Willert, M. Mönch, D. Jäckel, K. Leopold, U. Grittner, D. Nischk, S. Senner, P. Falkai, O. Pogarell, S. Sachenbacher, J. Gallinat, M. Lambert, A. Zimmermann, F. Bermpohl, MA. Daniels, A. Heinz, M. Schouler-Ocak, CU. Correll, PJ. Uhlhaas, A. Bechdolf

**Affiliations:** 1 Department of Psychiatry and Psychotherapy, Charité Campus Mitte, Charité Universitätsmedizin Berlin, Corporate Member of Freie Universität Berlin, Humboldt-Universität Zu Berlin, Berlin Institute of Health, Charitéplatz 1, 10117 Berlin, Germany; 2 Department of Psychiatry, Psychotherapy and Psychosomatics, Vivantes Klinikum Am Urban and Vivantes Klinikum Im Friedrichshain, Berlin, Germany; 3 Institute of Biometry and Clinical Epidemiology, Charité Universitätsmedizin Berlin, Corporate Member of Freie Universität Berlin, Humboldt-Universität Zu Berlin, Berlin Institute of Health, Charitéplatz 1, 10117 Berlin, Germany; 4 Department of Psychiatry and Psychotherapy, University Hospital Carl Gustav Carus, Technical University Dresden, Dresden, Germany; 5 Department of Social Psychiatry, Zentrum Für Psychiatrie, Reichenau, Germany; 6 Department of Psychiatry and Psychotherapy, LMU University Hospital, LMU Munich, Munich, Germany; 7 Max-Planck-Institute of Psychiatry, Munich, Germany; 8 German Center for Mental Health (DZPG) Site Munich/Augsburg, Munich, Germany; 9 Department of Psychiatry and Psychotherapy, University Medical Center Hamburg-Eppendorf, Hamburg, Germany; 10 Psychiatric University Clinic of Charité at St. Hedwig Hospital, Berlin, Germany; 11 Department of Psychiatry and Psychotherapy, University of Tübingen, Tübingen, Germany; 12 German Center for Mental Health (DZPG) Site Tübingen, Tübingen, Germany; 13 Department of Child and Adolescent Psychiatry, Charité Universitätsmedizin Berlin, Corporate Member of Freie Universität Berlin and Humboldt Universität Zu Berlin, Berlin, Germany; 14 Departments of Psychiatry and Molecular Medicine, Donald and Barbara Zucker School of Medicine at Hostra/Northwell, Hempstead, NY USA; 15 Department of Psychiatry, The Zucker Hillside Hospital, Northwell Health, Glen Oaks, NY USA; 16 Institute of Neuroscience and Psychology, University of Glasgow, Glasgow, UK; 17 German Center for Mental Health (DZPG) Site Berlin/Potsdam, Berlin, Germany

**Keywords:** Early psychosis, Early intervention, Supported employment and education, Individual placement and support

## Abstract

SEEearly is a multi-site randomized controlled trial (RCT) investigating the efficacy of supported employment and education (SEE) in adolescents and young adults aged 16 to 35 with early psychosis. In this publication we report baseline characteristics of the cohort with an emphasis on educational and vocational status at study entry. Between October 2022 and December 2024, a total of 184 participants with a diagnosis of schizophrenia spectrum disorder were recruited across six sites in Germany. At baseline, 53.3% were not engaged in employment, education, or training (NEET); only 20.1% were competitively employed and 26.6% were enrolled in education or training. A majority of the participants (97.8%) had a second or third level school-leaving qualification, yet only 39.1% of participants had completed post-secondary qualification indicating delayed or interrupted vocational trajectories. The sample is predominantly male (69.6%) and single (87.0%). Nearly 45% of participants had comorbid substance misuse. Functional assessments revealed marked psychosocial impairments, while ratings on symptom severity indicated moderate psychopathology. Compared to previous RCTs on SEE, the SEEearly sample is larger and more diagnostically homogenous. Baseline data show considerable disruptions in vocational trajectories at study entry. The findings highlight early and substantial deviations in vocational trajectories in young adults with early psychosis and underscore the need for interventions that target both educational and vocational attainment.

**Supplementary Information:**

The online version contains supplementary material available at https://doi.org/10.1007/s00406-026-02236-8.

## Introduction

The first onset of psychotic disorders usually occurs during adolescence and early adulthood [1, 2], a period during which individuals are typically developing their education, employment, and career trajectories. Low rates of secondary education completion, challenges in obtaining competitive employment (especially positions that require higher educational qualifications), and low vocational recovery rates reflect the chronic nature of the illness. These factors significantly contribute to the burden faced by individuals and their families, and to the overall costs that this illness imposes on society. Young people who are in treatment for psychotic disorders are three times more likely to affirm that they are not in education, employment, or training (NEET) than individuals in the general population [3, 4].

Early intervention programs that offer intensive, phase-specific psychosocial and pharmacological treatment within the first five years of an initial psychotic episode have been found to significantly improve outcomes and reduce negative consequences [5–8]. However, most early intervention programs for people experiencing early psychosis still focus on improving symptoms and preventing relapse, rather than targeting educational and vocational recovery [5, 6]. In recent years, there has been an increasing body of research on psychosocial interventions specifically targeting vocational recovery in people with severe mental illness (SMI). Based on over 30 randomized controlled trials, supported employment (SE), in keeping with the individual placement and support (IPS) model, has been found to be the most effective psychosocial approach for obtaining competitive employment in this population) [9–11]. Beyond vocational outcomes, SE and sustained competitive employment have been positively associated with clinical, social, and economic outcomes, as well as with improved quality of life [12–14]. Despite these positive findings, there have only been eight RCTs of SE in early psychosis to date [15–22]. While these studies underscore the substantial effectiveness of SE in early psychosis, they have mostly focused on employment status as a marker for vocational recovery and did not report on education status at baseline [15, 19, 23, 24]. These outcomes fail to capture the sequential nature of vocational trajectories, which include qualification completion, transition between educational and occupational roles and sustained active participation in education/employment. Christensen et al. [21] and Killackey et al. [16] applied SEE instead of SE, but their sample presented heterogenous with regard to diagnosis and showed high rates of NEET at baseline. Nuechterlein et al. [17] applied SEE to a sample that consisted only of participants with early psychosis, but their trial presented with a smaller sample size compared to the SEEearly trial.

The concept of vocational trajectories has been examined primarily in studies of the general population. These studies have shown that certain sociodemographic factors such as school-leaving qualification [25], gender [26] and social background [27] influence transition from school to vocational training/employment. Additionally, e*arly* career unemployment, in particular, was shown to be associated with an increased risk of longterm unemployment [28], whereas high level school-leaving qualifications significantly increase the likelihood of regular vocational training [27]. The impact of SMI, and early psychosis specifically, on milestones within vocational trajectories has not been examined in detail yet.

The present manuscript introduces the following conceptual considerations to SEE: a) recruitment of participants who are currently in mainstream education/employment to examine sustained attainment during follow-up, b) application of SEE rather than SE alone and c) examination of delayed or disrupted vocational trajectories at baseline and therefore d) proposition of education and employment as interconnected stages within the framework of vocational trajectories.

In the following we describe the sociodemographic, clinical, and psychometric characteristics of the study population at baseline with an emphasis on vocational trajectories and current engagement in mainstream education/competitive employment. Furthermore, we outline the recruitment process and data collection methods and compare our findings with prior research on SEE in early psychosis.

## Methods

The design and methodology of the SEEearly trial have been described in detail elsewhere [29]. Key aspects regarding setting, participants, recruitment, and data collection are summarized here.

The SEEearly trial is a six-site, prospective, rater-blinded, two-arm superiority RCT. We compare SEE + TAU to TAU alone in the respective recruitment centers over 12 months. The trial was evaluated and approved by the Ethics Committee at Charité Universitätsmedizin Berlin (EA2/168/22) and afterwards by all local ethics committees. The trial was registered in the national and international trial register DRKS (DRKS00029660) ahead of the start of recruitment.

### Participating sites

Recruitment took place in academic and non-academic clinical settings across Germany, involving the following outpatient units: *Vivantes Hospital Am Urban and Vivantes Hospital im Friedrichshain, Charité Universitätsmedizin Berlin*, Department of Psychiatry, Psychotherapy and Psychosomatic Medicine, Dieffenbachstraße 1, 10,967 Berlin, Germany, *Charité Universitätsmedizin Berlin* with a) Department of Child and Adolescent Psychiatry, Psychosomatic Medicine and Psychotherapy, Campus Virchow Klinikum, Augustenburger Platz 1, 13,353 Berlin, Germany, b) Department of Psychiatry and Psychotherapy, Campus Mitte, Charitéplatz 1, 10,117 Berlin, Germany, c) Psychiatric University Hospital at St. Hedwig’s Hospital, Große Hamburger Straße 5–11, 10,115 Berlin, Germany, *University Medical Center Hamburg-Eppendorf*, Department of Psychiatry and Psychotherapy, Martinistraße 52, 20,246 Hamburg, Germany, *LMU University Hospital, LMU Munich*, Department of Psychiatry and Psychotherapy, Nussbaumstraße 7, 80,336 Munich, Germany, *Zentrum für Psychiatrie Reichenau*, Department of Social Psychiatry, Feursteinstraße 55, 78,479 Reichenau, Germany, *Ulm University, District Hospital Gunzburg*, Department of Psychiatry II, Lindenallee 2, 89,321 Gunzburg/Ulm, Germany.

### Study population

The sample size calculation was based on the primary dichotomous outcome: "steady participation for at least 50% of the 12-month follow-up period in competitive employment and/or mainstream education." Prior studies reported rates between 39 to 44% for IPS and 11% to 23% for TAU [30–32]. To detect a difference of 24% between the two groups, with 40% in the IPS-arm versus 16% in the control-arm, the required sample size to find a significant effect with a power of 0.9 at a two-sided significance level of 0.05 is 144 participants (72 per treatment arm). The sample size calculation was based on a χ^2^-test. The power calculation was performed using software NQuery Version 8.4.1.0. Published drop-out and lost to follow-up rates in studies on SE and SEE range from 5% [33] to 30% [34]. Assuming a drop-out rate of 22%, as observed in a pilot RCT [22], the targeted number of participants to be recruited was 184.

Inclusion criteria were 1) adolescents and young adults aged 16–35 with 2) a clinical diagnosis of early psychosis (fulfilling DSM-5 schizophrenia spectrum or other psychotic disorder criteria and onset of psychosis within the last 5 years or initial presentation to mental health services due to psychotic symptoms within the last 5 years) [6]. Participants had to 3) express general interest in competitive employment and/or mainstream education and 4) show sufficient German language abilities (≥ A2). Exclusion criteria were 1) learning disability or mental retardation, 2) insufficient German language abilities (< A2) and 3) physical or organic handicap that seriously impedes functioning.

### Recruitment and randomization

Recruitment was conducted in the outpatient units of the participating sites from October 18, 2022, to December 18, 2024. Clinicians and/or psychologists referred patients who expressed interest in participating in the study to a research assistant who established contact with the patient to plan further appointments. Study information was available through posters and leaflets in waiting rooms and clinical units as well as on a website (www.seeearly.de).

Randomization was carried out using a computer-generated list with a 1:1 ratio. Since randomization took place after the baseline assessment, it is not described in detail here.

### Data collection and management

Baseline assessment was conducted face-to-face by a trained research assistant after participants were informed about the study and provided written informed consent. In the case of minors, parents/guardians were also informed and provided written informed consent. The data was then transferred to an eCRF in REDCap® (Research Electronic Data Capture, Long Term Support Version).

The baseline assessment included diagnoses according to DSM-5 (Diagnostic and Statistical Manual of Mental Disorders, version 5), sociodemographic information, including current employment/education and housing situation, prior psychiatric treatment, and motivation for change regarding employment or education. Additionally, the standardized instruments listed below were used to assess different dimensions of mental health:

1. Positive and Negative Syndrome Scale (PANSS) [35] for *psychopathology*
2. International Classification of Functioning, Disability, and Health (Mini-ICF-APP; [36] and Global Assessment of Functioning (GAF) [37] for *functional impairment*
3. Modified version of the Addiction Severity Index (ASI) [38] for *substance use* and Daily Sessions, Frequency, Age of Onset and Quantity of Cannabis Use Inventory (DFAQ-CU) [39] for *cannabis use* specifically
4. Short version of the World Health Organization Quality of Life scale (WHOQOL-BREF) [40] for *subjective quality of life*

Inter-rater reliability for the PANSS was monitored during the study using an internationally standardized clinical case video demonstration. The intraclass correlation coefficient for ordinal data as well as the weighted inter-rater reliability measures Krippendorff’s Alpha and Gwet’s AC2 yielded moderate to good values on Altmann benchmark range (ICC = 0.62, 95%-bootstrap-CI: 0.62 to 0.78; Alpha = 0.51, 95%-CI: 0.35 to 0.68; AC2: 0.60, 95%-CI: 0.49 to 0.71).

### Data analysis

The statistical analyses were performed using R software (version 4.2.2) and R Studio software (version 2023.03.1 build 446) [41]. Patient characteristics are summarized without group stratification by mean and standard deviation (SD) for interval data, median and interquartile-range (IQR), minimum and maximum for skewed interval and ordinal data, and absolute and relative frequencies for categorical data. Scores (i.e. PANSS, QoL, Mini-ICF) were aggregated by appropriate formulas and summarized as data above. To enhance comparability with other studies, the total PANSS score was converted to the total Brief Psychiatric Rating Scale (BPRS) using a fitted linear model based on reference data [28]. To compare SEEearly sample characteristics with those of other trials, pairwise standardized mean differences (SMD) were calculated for age, total BPRS, gender, partnership status, primary diagnosis, employment/education status at intake, and educational qualification. The SMD is a standardized effect size which is Cohen’s d in case of continuous measures with normal distribution. Extensions to multinomial and binary variables as proposed by Austin 2009 and Yang & Dalton 2012 are used depending on the scaling [5, 45]. The average SMD and its 95% confidence interval were then extracted for each characteristic.

## Results

### Sample characteristics

**Fig. 1:**
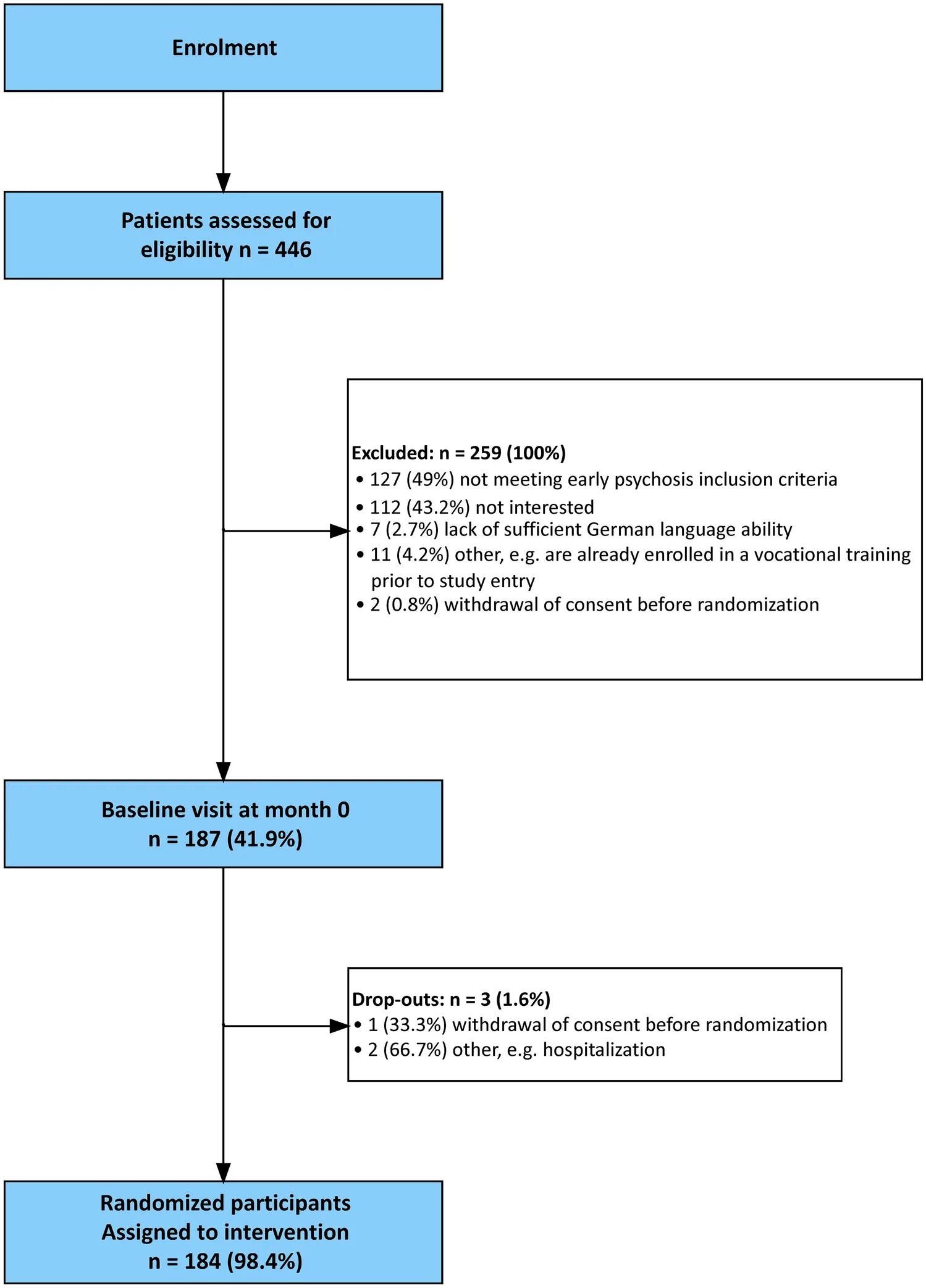
Flowchart of the SEEearly study until randomization

From October 18, 2022, to December 18, 2024, 446 individuals were screened for eligibility. Out of these 446, 259 (58%) were excluded. Reasons for exclusion were i) not meeting early psychosis criteria (n = 127; 48.5%), ii) lack of interest in participation (n = 112; 42.7%), iii) not meeting language ability criteria (n = 7; 2.7%) and iv) enrollment in vocational training (n = 13; 5%) (see Fig. 1). Of the recruited population 98.4% (n = 184) were randomized after baseline visit in month 0. Only 3 participants (1.6%) withdrew consent before randomization. Thus, the recruited sample is relatively representative of young people with EP and the desire to work or study.

**Fig. 2:**
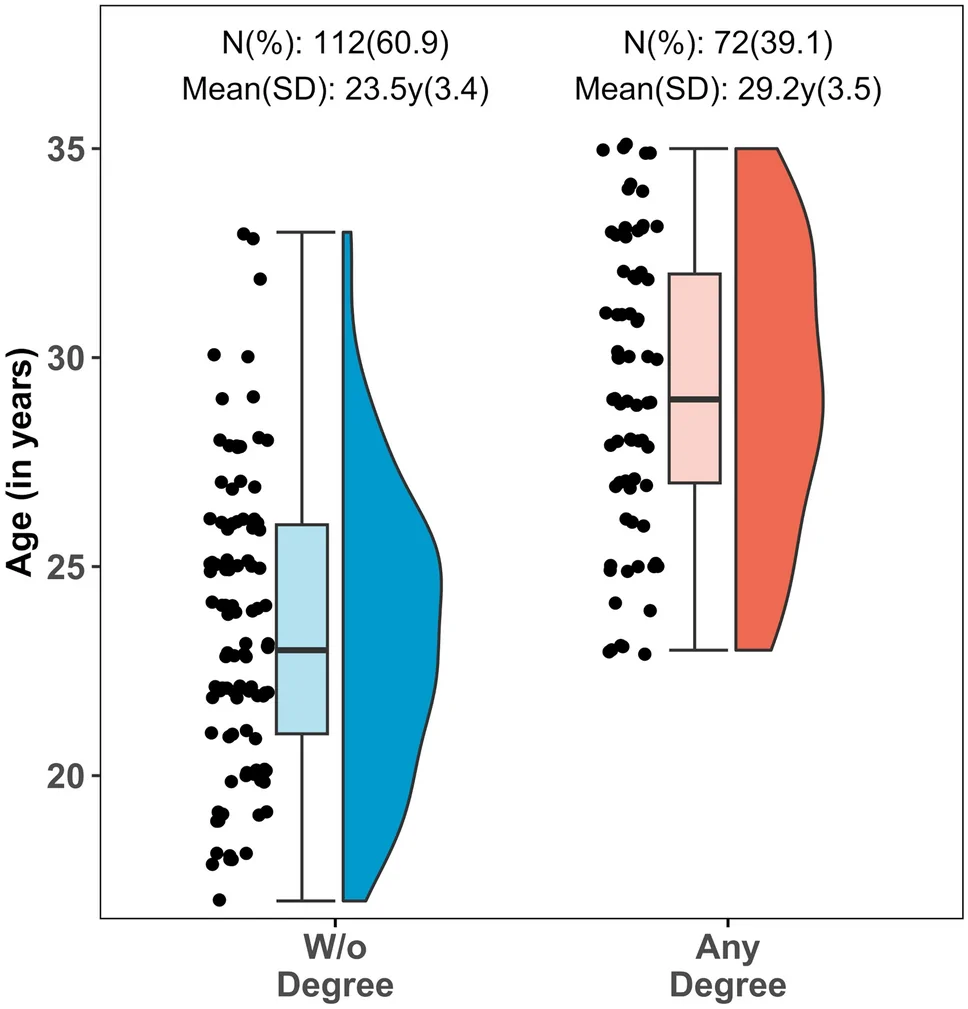
Raincloud plots to visualize the age distribution among SEEearly study participants per presence of any degree of vocational training or higher education. W/o without, SD standard deviation, y years

Table 1 includes sociodemographic variables as well as employment-/education-related variables. The majority of the cohort were male (n = 128; 69.6%) and single (n = 160; 87%). About half of the participants were foreign born or had at least one foreign born parent (n = 85, 46.1%). At baseline, 6.180 participants (97.8%) had a second or third level school-leaving qualification. This is equivalent to the International Standard Classification of Education (ISCED) level two (generally ninth or tenth grade) or level three (generally twelfth or thirteenth grade). Further, 2.2% had no school-leaving qualification (ISCED-A 030 or 1). The highest education category, i.e. having completed vocational training or higher education, was met by 39.1% (n = 72). 20.1% (n = 37) of participants were in competitive employment and 26.6% (n = 49) in education/training at study entry. Additionally, 53.3% of participants were not enrolled in education or training (NEET). To examine potential delays in vocational trajectories, we tested for age distribution in participants with a degree compared to those without a degree. Those with a degree presented a higher mean age than those without any degree (29.2 years vs. 23.5 years) (see Fig. 2).

**Table 1:**
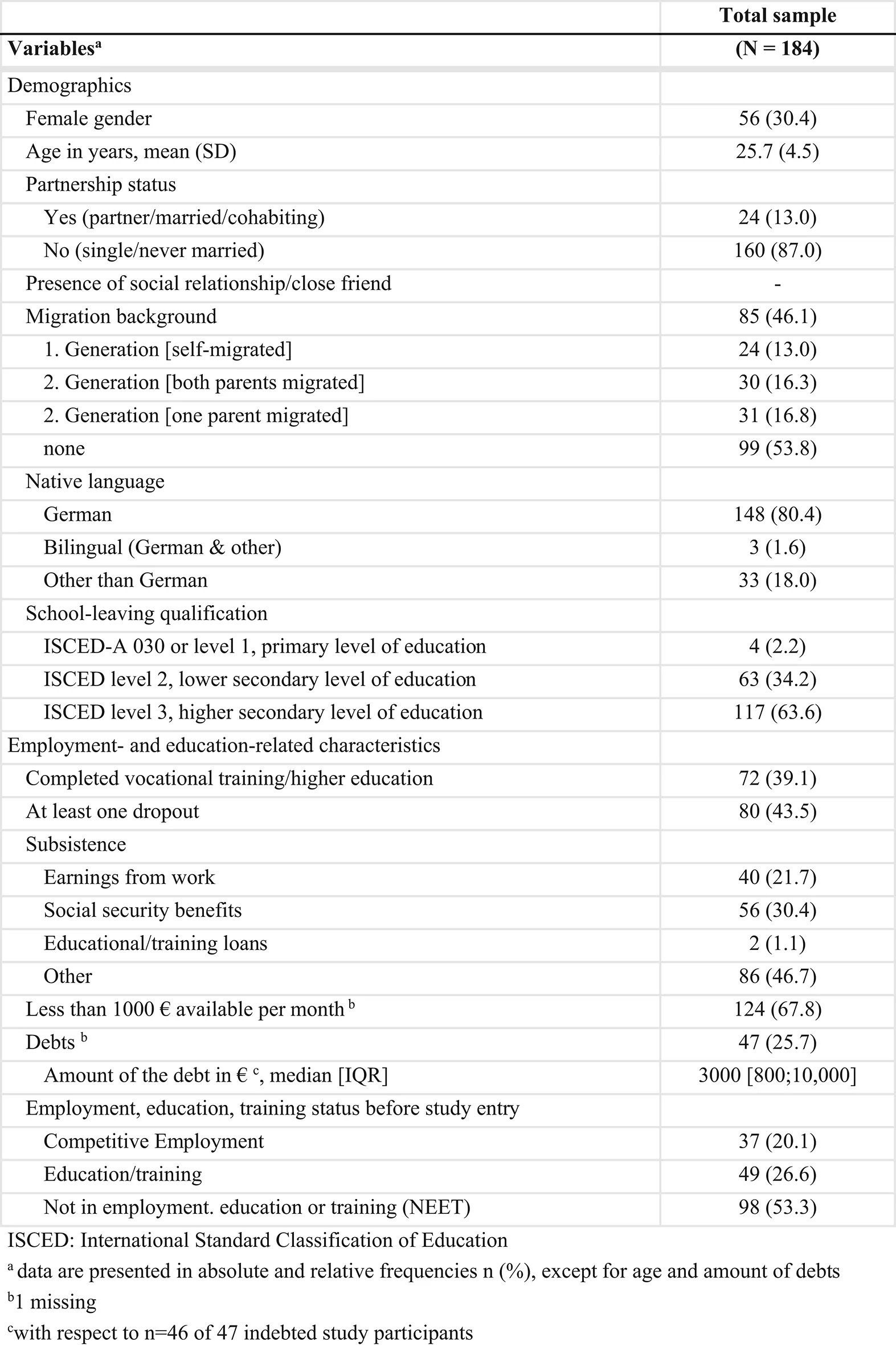
SEEearly – Baseline Sociodemographic and employment-/education-related variables:

With regards to financial support, 21.7% (n = 40) earned income from competitive employment, 30.4% (n = 56) received social security benefits, including educational loans, while most participants received financial support from alternative sources, such as family members (n = 86, 46.7%). About two-thirds of participants (67.8%) lived below the poverty line, as defined as having less than 1000 Euros per month available for sustenance.

Table 2 covers details on diagnosis and functionality. All participants had a diagnosis of early psychosis according to the guidelines established by Bird et al. [6]. The most frequent psychotic disorders were 1) schizophrenia (52.7%), 2) brief psychotic disorder (16.8%), and 3) schizoaffective disorder (14.7%). Additionally, 44.6% of the participants presented with comorbid substance misuse.

**Table 2:**
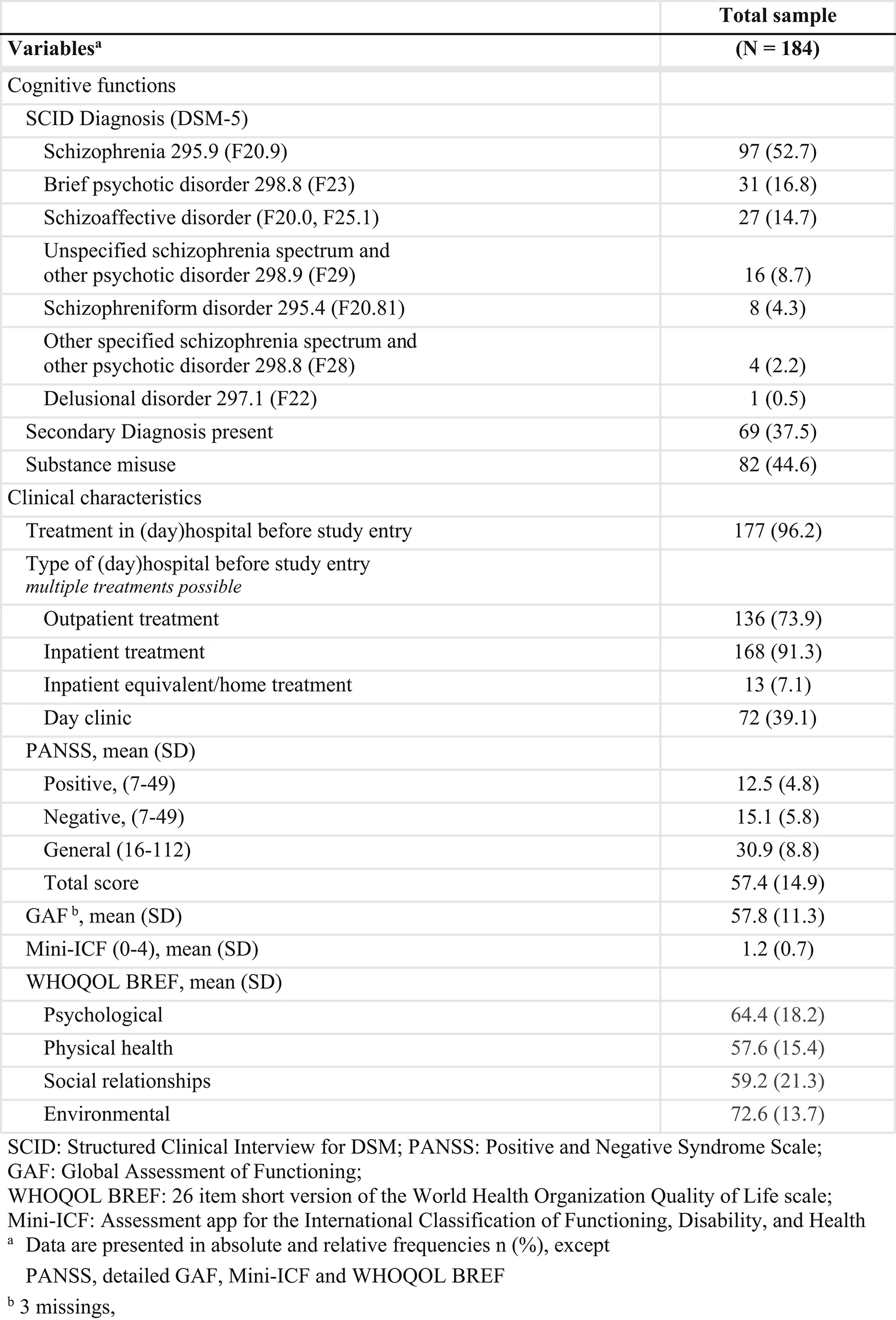
SEEearly – Baseline: details on diagnosis and functionality

### Positioning in context of prior trials

Table 3 describes the sample characteristics of the SEEearly sample in comparison to earlier trials on SE and SEE in young adults. Across the earlier studies, a heterogeneity in sample characteristics is highlighted. Compared to prior trials, the SEEearly population showed substantial differences in primary diagnosis (mean SMD: 0.81; 95%-CI: 0.78–0.84) and educational qualification (mean SMD: 1.07; 95%-CI: 1.03–1.11). Specifically, all SEEearly participants (100%) were diagnosed with a schizophrenia spectrum disorder according to DSM 5 criteria, and 63.6% had achieved an ISCED level 3 educational qualification. This demonstrates much higher homogeneity in these areas compared to most other randomized controlled trials (RCTs). Moderate heterogeneity was observed between studies regarding age (mean SMD: 0.64; 95%-CI: 0.62–0.67) and BPRS scores (mean SMD: 0.64; 95%-CI: 0.60–0.67). The SEEearly population was, on average and compared to the IPS AT population [22], older (mean 25.7 years vs. 26.3 years) and had a higher mean BPRS score (31.7 vs. 27.42). However, the mean BPRS value in SEEearly was lower than that of other samples. Minor sample heterogeneity was found for sex, partnership status, employment, and education, though SEEearly participants had higher rates of employment and educational enrolment.

**Table 3:** SEEearly – Demographics of the SEEearly sample in comparison to earlier trials on SE and SEE in young adults

	**SEEearly** **2025**		**Killackey** **2008 **[15]	**Bond** **2016 **[19]	**Killackey** **2019 **[16]	**Christensen** **2019 **[21]	**Sveinsdottir** **2019 **[20]	**Nuechterlein** **2020 **[17]	**Erickson** **2020 **[18]	**IPS-AT** **2025 **[22]	**Statistics**^**b**^
Variables^**a**^	**(N = 184)**		**(N = 41)**	**(N = 109)**	**(N = 146)**	**(N = 720)**	**(N = 96)**	**(N = 69)**	**(N = 109)**	**(N = 94)**	**SMD** **(95% CI)**
Demographics											
Gender, male	128 (69.6)		34 (83)	75 (68.8)	101 (69.2)	444 (61.7)	65 (68)	46 (67)	90 (82.6)	51 (43.6)	0.25 (0.24;0.26)
Age, mean (SD)	25.7 (4.5)		21.4 (2.3)	25.96 (2.75)	20.4 (2.4)	32.8 (9.9)	23.9 (3.3)	24.5 (4.1)	23.1 (3.4)	26.3 (4.8)	0.64 (0.62;0.67)
Partnership status Yes (partner/married/cohabiting) No (single/never married) Missing	24 (13.0) 160 (87.0)		8 (21.6) 29 (78.4) 4	2 (1.8) 107 (98.2)	4 (2.7) 142 (97.3)	142 (19.7) 578 (80.3)	-/NA -/NA	18 (26.1) 51 (73.9)	-/NA -/NA	18 (19.1) 76 (80.9)	0.28 (0.27;0.29)
Primary diagnosis Psychotic disorder (F2x.x) Mood disorder (F3x.x) Other SIPD (F1.x.x) acc. ICD	184 (100) - - -		41 (100) - - -	51 (46.8) 50 (45.9) 8 (7.3) -	100 (68.5) 37 (25.3) 9 (6.2) -	551 (76.5) 169 (23.5) - -	-/NA -/NA -/NA -/NA	69 (100) - - -	72 (66) 30 (28) 7 (6) -	60 (63.8) 11 (11.7) - 23 (24.5)	0.81 (0.78;0.84)
BPRS total^c^, mean (SD)	31.7 (8.6)			33.01 (8.54)	45.5 (12)				37.13 (7.96)	27.42 (9.18)	0.64 (0.60;0.67)
Quality of Life Instruments	**WHOQOL BREF** Psychological 64.4 (18.2) Physical health 57.6 (15.4) Social relations 59.2 (21.3) Environmental 72.6 (13.7)		**QoLS:** 75.3 (16.4)	-/NA	**QoL:** Psychological 64.7 (16.1) Physical health 53.4 (19.6) Social relations 57.4 (21.9) Environmental 62.0 (16.0)	-/NA	**Global** **well-being** 4.9 (1.8)	-/NA	-/NA	**G-factor:** 5.8 (1.7)	-
Employment, education, training status at intake											
In employment	37 (20.1)		3 (7.3)	-	24 (8.2)	-	-	14 (20.3)	11 (10.1)	13 (13.8)	0.26 (0.25;0.27)
In education/training	49 (26.6)		-/NA	-/NA	26 (17.8)	-/NA	-/NA	13 (18.8)	-/NA	17 (18.1)	0.37 (0.35;0.40)
Other	98 (53.3)		-/NA	-/NA	108 (74.0)	-/NA	-/NA	42 (60.9)	-/NA	64 (68.1)	
School-leaving qualification											
ISCED-A 030 or level 1	4 (2.2)		13 (36.1)	32 (29.4)	33 (22.6)	279 (28.8)	38 (39.6)^*^	-/NA	-/NA	2 (2.1)	1.07 (1.03;1.11)
ISCED level 2	63 (34.2)		23 (63.9)	51 (46.8)	54 (37)	175 (24.3)	-/NA	-/NA	-/NA	14 (14.9)	
ISCED level 3	117 (63.6)		0	26 (23.9)	59 (40.4)	266 (36.9)	-/NA	-/NA	-/NA	78 (83)	

*SIPD*: Substance-Induced Psychotic Disorder; *ICD*: International Statistical Classification of Diseases and Related Health Problems;

*BPRS*: Brief Psychiatric Rating Scale; *ISCED*: International Standard Classification of Education; *QoLS*: 21 item Quality of Life Scale; *QoL*: Quality of Life;

G-Factor: QoL dimension “life in general”, range: 1–10; WHOQOL BREF: 26 item short version of the World Health Organization Quality of Life Scale;

Global well-being: 10-point Cantril Ladder Scale for current situation

^a^ Data are presented in absolute and relative frequencies n (%), except age, total BPRS and quality of life instruments

^b^ Potential differences between SEEearly sample and other samples were examined using the mean of all pair-wise standardized mean differences for continuous and categorical variables

^c^ BPRS for SEEearly and IPS-AT sample were translated from PANSS total score using a fitted linear model based on reference data (formula: $\mathrm{BPRS}=- 1.51+0.58\times \mathrm{PANSS}$)

^*^ omitted for SMD-calculation

With respect to quality-of-life assessments the SEEearly sample had a mean WHOQOL BREF value of 64.4 (sd = 18.2) in the psychological domain, a mean value of 57.6 (sd = 15.4) in the physical health domain, a mean value of 59.2 (sd = 21.3) in the social relationship domain and 72.6 (sd = 13.7) as mean value for the environmental domain.

## Discussion

Compared to previous RCTs on SE and SEE in young adults, our study included the largest sample to date and applies the strictest inclusion criteria regarding diagnosis. While most RCTs included both psychotic disorders and mood disorders [16, 18, 19, 21, 22], the sample in Nuechterlein et al. [17] and Killackey et al. [15] consisted only of participants with a diagnosis of psychotic disorder according to DSM 5. However, these studies present a small sample size of 41 [15] to 69 [17]. While it could be argued that, particularly in the early stages of mental illness, there may be changes in diagnosis during the course of illness, the SEEearly sample, by fulfilling criteria for only schizophrenia spectrum disorder, presents a population with a higher risk of serious mental illness (SMI) compared to prior RCTs, which also included affective disorders. Overall, the SEEearly sample showed comparable baseline rates of participants in competitive employment but significantly higher rates of participants in mainstream education when compared to the previously mentioned RCTs. Prior research has demonstrated unemployment rates up to 33% in first episode psychosis [42] with unemployment being the most contributing factor to indirect costs of psychotic illnesses [43, 44]. Regarding the design of the SEEearly trial, we deliberately also included participants with existing employment and/or training as both obtaining and maintaining an existing job/training is relevant for the overall outcomes and functional recovery.

It should be noted that in comparison to prior trials a very high proportion of participants of the SEEearly trial obtained second or third level school-leaving qualifications (98.4%). However, only 39.1% completed post-secondary qualification compared to 81% of young adults aged 20 to 34 in the general population [45]. Furthermore, participants with post-secondary qualification were significantly older than participants without a degree. Since it could be argued that participants have not reached the stage of completion of education yet, we additionally tested for age distribution against education/employment status in both groups. Fig. S1 shows that among those without any degree, but also among those with obtained secondary qualification almost half belong to the NEET group. Additionally, the sample presented high rates of at least one drop-out from secondary education, regardless of educational qualification.

Taken together, our findings indicate disruptions and delay in vocational trajectories despite overall good school-leaving qualifications. Within the framework of vocational trajectories, educational disruptions, such as delayed training entry or drop-out events, appear early, shape later competitive employment outcomes and may serve as an early marker of functional impairment. Prior studies have already shown that serious mental illness (SMI) is an important factor in why young people do not complete (secondary) education and how the lack of integration into regular work and education, in turn, has a negative impact on mental health [46, 47]. Additionally, employment rates have been found to be significantly lower already in young people at ultra-high risk for psychosis compared to the general population and also negatively correlate with the likelihood of a subsequent diagnosis [48]. It is therefore necessary to provide meaningful and effective interventions that go beyond mere symptom control for those affected by early symptoms of potential SMI. Regarding SE, focusing only on competitive employment seems inadequate, as a lack of educational qualifications is a significant barrier to obtaining competitive employment—beyond just precarious temporary jobs.

### Limitations

This study has several limitations that should be taken into account when interpreting the findings. First, as in all RCTs, the generalization of the results to the population outside of the randomized sample is limited. However, since more than 50% of the eligible sample agreed to be randomized, the generalizability of the results is very high compared with RCTs in other areas. Second, there is a pronounced overrepresentation of metropolitan areas among study sites, with rural regions significantly underrepresented. Third, no study site was located in the eastern federal states of Germany („neue Bundesländer “). Both limit the geographic and sociocultural representativeness of the sample. Fourth, the conversion between PANSS and BPRS scores was based on estimation methods subject to measurement error. These approximations may introduce noise into symptom ratings and limit the comparability of symptom severity across studies. Finally, since IPS follows the criterion of “zero exclusion”, the described sample constitutes of a self-selected group of participants with early psychosis who subjectively reported limitations in mainstream education and/or competitive employment.

## Conclusion

Overall, the SEEearly sample is well comparable with other trials of SEE in young people with mental disorders. Along with Nuechterlein et al. [17] and Killackey et al. [15], the SEEearly trial includes participants with a diagnosis of schizophrenia spectrum disorder only and presents with a sample size that has the potential to overcome prior methodological limitations. Our findings from baseline characteristics underscore the clear need for targeted and effective interventions, such as IPS, that support the attainment and maintenance of vocational qualifications. The framework of vocational trajectories offers a more nuanced and detailed picture and acknowledges that disruptions in earlier stages may shape later vocational capacity.

## Supplementary Information

Below is the link to the electronic supplementary material.Supplementary file1 (TIF 29124 KB)

## Data Availability

The project leader and the project group will have access to the final dataset. The dataset generated during the current study will not be publicly available, as data sharing was not included in the protocol and consent form on which the ethical approval was based.
